# Classification of chronic myeloid leukemia cell subtypes based on microscopic image analysis

**DOI:** 10.17179/excli2019-1292

**Published:** 2019-06-14

**Authors:** Narjes Ghane, Alireza Vard, Ardeshir Talebi, Pardis Nematollahy

**Affiliations:** 1 Department of Bioelectrics and Biomedical Engineering, School of Advanced Technologies in Medicine and Student Research Center, Isfahan University of Medical Sciences, Isfahan, Iran; 2 Department of Bioelectrics and Biomedical Engineering, School of Advanced Technologies in Medicine and Medical Image and Signal Processing Research Center, Isfahan University of Medical Sciences, Isfahan, Iran; 3 Department of Pathology, School of Medicine, Isfahan University of Medical Sciences, Isfahan, Iran

**Keywords:** Chronic Myeloid Leukemia (CML), blood cancer, microscopic image processing, classification, decision tree classifier

## Abstract

This paper presents a simple and efficient computer-aided diagnosis method to classify Chronic Myeloid Leukemia (CML) cells based on microscopic image processing. In the proposed method, a novel combination of both typical and new features is introduced for classification of CML cells. Next, an effective decision tree classifier is proposed to classify CML cells into eight groups. The proposed method was evaluated on 1730 CML cell images containing 714 cells of non-cancerous bone marrow aspiration and 1016 cells of cancerous peripheral blood smears. The performance of the proposed classification method was compared to manual labels made by two experts. The average values of accuracy, specificity and sensitivity were 99.0 %, 99.4 % and 98.3 %, respectively for all groups of CML. In addition, Cohen's kappa coefficient demonstrated high conformity, 0.99, between joint diagnostic results of two experts and the obtained results of the proposed approach. According to the obtained results, the suggested method has a high capability to classify effective cells of CML and can be applied as a simple, affordable and reliable computer-aided diagnosis tool to help pathologists to diagnose CML.

## Introduction

White blood cells (WBCs) are types of immune cells that help fight against infections and other diseases. They are typically divided into two main groups: 1- Granulocytes (include Neutrophils, Eosinophils and Basophils); 2- Agranulocytes (include Lymphocytes and Monocytes). Extracting information from WBCs can assist pathologists in Leukemia diagnosis (Mohan, 2015[17]). Leukemia is a type of cancer that commonly initiates in the bone marrow and causes a high numbers of abnormal white blood cells (Bain, 2008[4]). 

According to the French-American-British (FAB) classification, leukemia; depending on type of effective WBCs, is divided in two groups: 1- myeloid and 2-lymphocytic (Coppola, 2010[6]). These two groups are the further sub-classified into acute and chronic types. The acute leukemia progresses more rapidly than chronic leukemia and requires immediate treatment. In summation, the four main types of leukemia are: Acute Lymphocytic Leukemia (ALL), Acute Myelogenous Leukemia (AML), Chronic Lymphocytic Leukemia (CLL) and Chronic Myelogenous Leukemia (CML). CML is a type of leukemia that affects the blood and bone marrow. This disease usually occurs after adulthood and rarely occurs in childhood. Although the initial diagnosis of chronic leukemia should include microscopic analysis of the bone marrow, the final diagnosis of CML is done based on the investigation of a peripheral blood smear under a microscope. In CML, the bone marrow generates too many abnormal white cells, called myeloid series (CML cells). The developmental stages of the CML cells, from immature to mature include: Myeloblasts, Promyelocytes, Myelocytes, Metamyelocytes, Bands and Neutrophils (Zittoun et al., 1994[38]). The characteristics of all types of CML cells are summarized in Table 1(Tab. 1). Myeloblasts are the most immature type of myeloid cells. The nuclei in these cells are large with a round or oval shape, fine chromatin and can have several nucleoli, whilst, the cell's cytoplasm is basophilic, and lacking in granules. Additionally, the area of the nucleus to the area of the cytoplasm ratio (N:C) is more than 80 % in Myeloblasts.

Promyelocyte cells are a little bigger than Myeloblasts with round or oval shaped nuclei, smooth chromatin and the possible presence of nucleoli. The cytoplasm's of promyelocytes are blue with numerous azurophilic granules; and the N:C is more than 70 %.

Myelocytes are typically smaller in size compared to Promyelocytes. Their nuclei are round to oval and lack the presence of a nucleolus. The chromatin in Myelocytes is comparably coarser than the chromatin in Promyelocytes and the cytoplasm of Myelocytes are typically light blue in color with secondary granules. Furthermore, the N:C in Myelocyte is more than 60 %.

Metamyelocytes are considerably smaller than Myelocytes with a kidney shaped nucleus and partly dense chromatin. They have a pink cytoplasm with numerous secondary granules and a N:C of more than 40 %.

Band cells have even less size than a Metamyelocytes, a curved and elongated nucleus with parallel sides and coarse chromatin, cytoplasm's similar to Metamyelocytes and a N:C typically between 30 % and 40 %.

Finally, Neutrophils are the most mature type of cells in the myeloid series that have lobulated Nuclei and dark purple stains with large dense chromatin. The cytoplasm's of Neutrophils are pale pink/tan with fine pink purple granules and a N:C between 20 % and 30 %.

The screening of prepared blood samples for cell counting and classification conducted by a pathologist is currently the main action taken in the diagnosis leukemia. This manual process is however; tedious, slow, time-consuming, and is largely dependent on experienced experts in this field (Roussel et al., 2010[26]). Therefore, computer-aided methods that autonomously partially or fully perform some steps of this process, can be very useful and helpful for experts and researchers (Fatichah et al., 2012[7]).

In the past years, various computer-aided approaches based on image processing methods have been presented to classify five types of mature WBCs (Neutrophils, Eosinophils, Basophils, Lymphocytes and Monocytes) (Sarrafzadeh et al., 2014[29]; Habibzadeh et al., 2012[12]; Putzu et al., 2014[21]) six types of immature WBCs (CML cells; Myeloblast, Promyelocyte, Myelocyte, Metamyelocyte, Band and Neutrophil) (Theera-Umpon and Dhompongsa, 2007[33]; Ramirez-Cortes et al., 2010[22]; Theera-Umpon, 2005[32]) plasma cells in myeloma (Saeedizadeh et al., 2016[28]) and effective cells in diagnosis other leukemia (CLL (Alférez et al., 2014[3]), AML and ALL (Laosai and Chamnongthai, 2014[15]; MoradiAmin et al., 2016[19])). 

In all these image processing methods for cell classification, usually a four-stage procedure has to been implemented. The four stages are: 1-preprocessing, 2-segmentation, 3-feature extraction and 4-classification. The main tasks of these stages and some image processing method used in the previous work are summarized as follows:

- Preprocessing: this stage is designed to enhance contrast of cells rather than background; while reduce noise and clutter of images. To contrast enhancement (Gonzalez and Woods, 2008[11]), techniques such as histogram stretching or equalization (Sabino et al., 2004[27]) and Gram-Schmidt orthogonalization (Tabrizi et al., 2010[31]) have been used. In others, to reduce noise, various image denoising methods such as median filter (Ghane et al., 2017[9]; Ross et al., 2006[25]) and Wiener filter have been utilized.
- Segmentation: in this stage, image segmentation algorithms and methods such as thresholding (Wu et al., 2006[36]), watershed (Ghane et al., 2017[9]; Jiang et al., 2003[14]), or active contour models (Zamani and Safabakhsh, 2006[37]; Vard et al., 2008[34]) are applied semi- or full-automatically to extract cells from background. These methods are also employed to separate cell components, nucleus and cytoplasm, from each other.
- Feature extraction: after segmentation, for each extracted cell, suitable image features that are able to discriminate each group of cells, are extracted from the cell, its nucleus and/or cytoplasm. The main image features usually utilized in previous work include: geometric features such as area, perimeter, compactness and eccentricity (MoradiAmin et al., 2016[19]); statistical information such as mean, standard deviation and skewness; color, such as RGB and L*a*b*(Sarrafzadeh et al., 2014[29]) and texture features such as Gray-Level Co-Occurrence Matrix (GLCM) (Sabino et al., 2004[27]) and local binary pattern (LBP)) (Agaian et al., 2014[2]).
- Classification: in the final stage, classification methods such as SVM (Putzu et al. 2014[21]; Tabrizi et al., 2010[31]), Random Forest (RF) (Mishra et al., 2017[16]) and Feed-forward Neural Networks (FFNN) (Theera-Umpon and Dhompongsa, 2007[33]) are used on the set of calculated features in the previous stage to determine the type of each cell.

For example (Theera-Umpon and Dhompongsa, 2007[33]), the classification of CML cells (Myeloblasts, Promyelocytes, Myelocytes, Metamyelocytes, Bands and Neutrophils) have been considered. In this work, nuclei of WBCs have been manually segmented. Next, texture and geometric features, based on the pattern spectrum and the area, have been extracted from them. Finally, ANN (Artificial neural network) was applied to classify each cell into the six groups of CML cells. This algorithm has been tested on 431 cells and the average of accuracy, reported in this paper, has been about 77 %.

Ramirez-Cortes et al. (2010[22]) also conducted the segmentation of WBCs manually for classification of the six groups of CML cells, followed by the extraction of some texture and geometric features including pattern spectrum, normalized area, and nucleus to cytoplasm area ratio from 54 nuclei of WBCs. In this paper, the average of accuracy, 87.6 %, has been obtained by a Feed-forward Neural Networks (FFNN) classifier as the best result.

Most of the previous studies for classification of WBCs based on image processing methods have some issues and limitations. For example, some of these approaches need to calculate many image features that increase diagnostic time (Putzu et al., 2014[21]). Some of them (Ramirez-Cortes et al., 2010[22]; Saeedizadeh et al., 2016[28]) have only been evaluated on a few numbers of images or cells and sometimes the reported results of them did not have high performance (Theera-Umpon and Dhompongsa, 2007[33]). In addition, most of these approaches have just been evaluated and compared only by opinion of one expert (Sarrafzadeh et al., 2014[29]; Habibzadeh et al., 2012[12]; Putzu et al., 2014[21]; MoradiAmin et al., 2016[19]).

In this paper, we propose a simple and efficient method based on image processing techniques for classification of effective cells in CML (Myeloblast, Promyelocyte, Myelocyte, Metamyelocyte, Band and Neutrophil) from microscopic images of both bone marrow and peripheral blood. In the proposed method, after automatic segmentation of WBCs, the cells and their nuclei are extracted. Then, a new combination of features based on three typical features and three new features are extracted from them. Finally, a novel decision tree is implemented for classification of CML cells. The main contribution of this paper is as follows:

- Database: in this project, 540 images have been gathered from 25 patients and 1730 WBCs have been extracted and classified from these images.
- Feature Extraction: three new geometric features have been calculated and extracted from WBCs that include: 1- Minimum thickness of Nucleus Mask to minimum convex thickness of Nucleus Mask ratio, 2- Minimum thickness of Nucleus Mask to Hausdorff distance between the nucleus and cell ratio and 3- Perimeters of smaller nucleus after splitting.
- Classification: a simple and efficient decision tree classifier has been introduced to classified CML cells to the eight groups (including two extra groups added to the six main groups as overlapping complementary groups).
- Validation: In this study, WBCs have been labeled by two pathologists in different times. Then, in order to assess proposed method, different evaluation parameters have been calculated to compare the results of our algorithm with diagnostic results of two experts.

The remainder of this paper is organized as follows: In “MATERIALS AND METHODS” Section, the proposed method is introduced. Experimental data and validation methods are explained in ''EXPERIMENTAL DATA AND VALIDATION METHODS'' Section. The experimental results are presented and analyzed in ''RESULTS AND DISCUSSION'' Section. Finally, the paper is concluded in ''CONCLUSIONS'' Section.

## Materials and Methods

**Figure 1:**
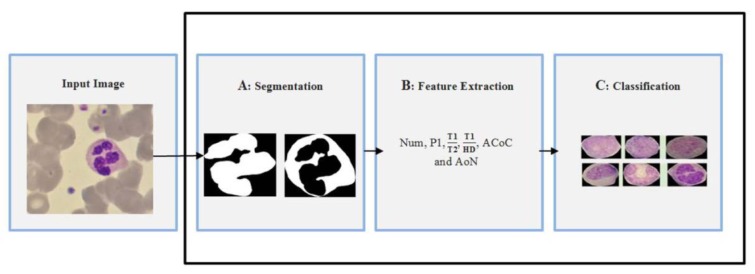
Block diagram of the proposed method

Classification of WBCs is one of the fundamental tasks in the microscopic image analysis. To classify WBCs of CML cells, we propose a three-part algorithm including automatic segmentation, feature extraction and classification. A schematic block diagram of the proposed method is presented in Figure 1(Fig. 1) and each part of this algorithm is described in the following of this section.

### A: Segmentation 

**Figure 2:**
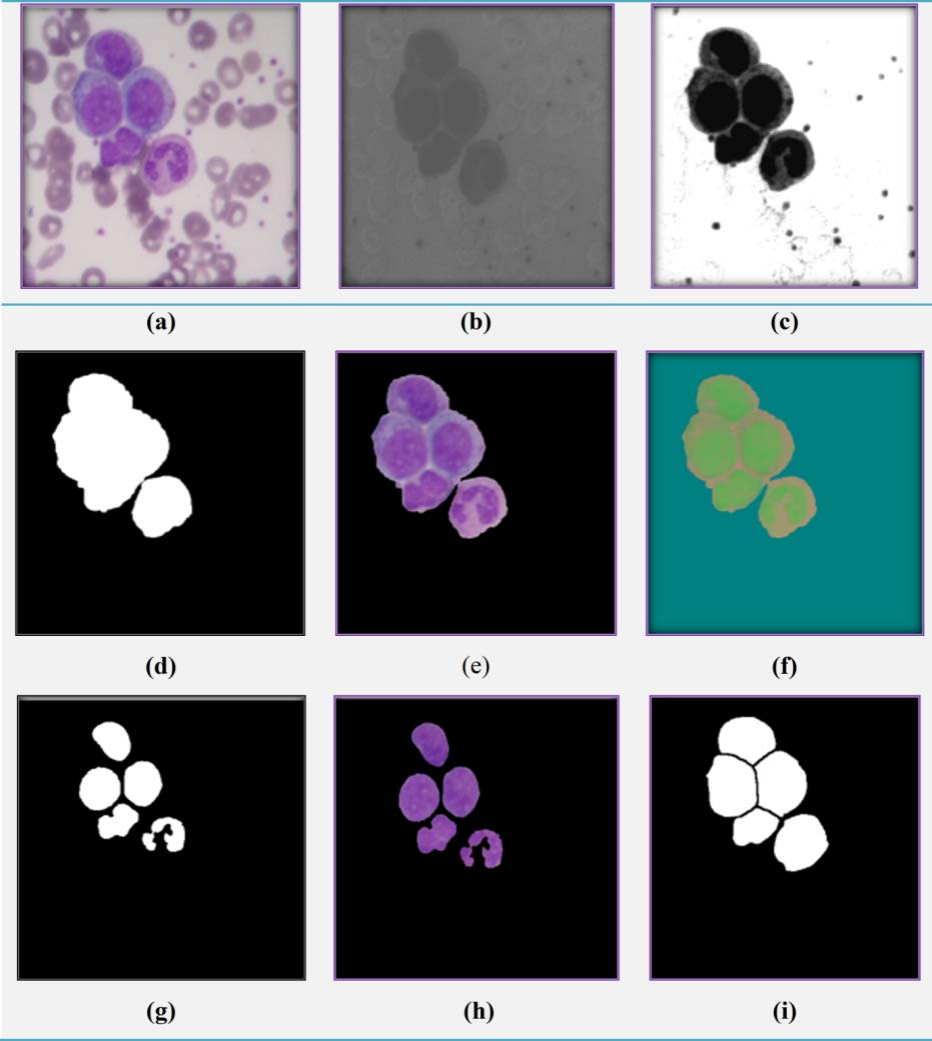
Results of automatic segmentation: (a) Original RGB image; (b) Y sub-band; (c) filtered image of EI Image; (d) Binary image of cells after thresholding and applying morphological operations; (e) RGB image of Cells; (f) L*a*b* image; (g) Binary image of nuclei (Nucleus Mask) after applying morphological operations; (h) the RGB image of Nucleus Mask;(i) output of watershed on (d) (Mask Cell).

In order to extract cells and segment nucleus and cytoplasm of them, we apply a simple and efficient method introduced in our previous work (Ghane et al. 2017[9]). In this method, to increase the contrast of the image, it is converted from RGB color space (Figure 2a(Fig. 2)) to CMYK color space and the Y component (Figure 2b(Fig. 2)) of it is enhanced as follows:


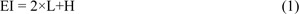


where EI denotes the enhanced image, L and H are linear contrast stretching and histogram equalization of Y component respectively. After enhancing the contrast (Figure 2c(Fig. 2)), a 3×3 minimum filter is employed on this image three times to decrease noise and clutter of the image. Next, the Otsu (1979[20]) thresholding method is used to extract binary mask of WBCs, and then some morphological operations including opening followed by closing and fill holes (Gonzalez and Woods, 2008[11]) are performed on the binary mask of cells to clean up it (Figure 2d(Fig. 2)). This binary mask is multiplied to the main RGB image and a cell image in this color space is obtained (Figure 2e(Fig. 2)). Since the components of RGB image are highly correlated, the cell image is converted from RGB color space to L*a*b* color space (Figure 2f(Fig. 2)). L*a*b* color system has three independent components that the intensity is represented by lightness (L*) and two color components are denoted by (a*) and (b*). As a cell's nucleus can be better discriminated from its cytoplasm in the a* component rather than two other components in L*a*b* color space, we select this component, and then employ a Gaussian filter with standard deviation, σ = 33, on it to decrease noise and clutter of the image. Next, in order to extract nucleus, a suitable normalization method (Rezatofighi and Soltanian-Zadeh, 2011[23]) is applied on the filtered image. After that, again the Otsu (1979[20]) thresholding method is used to extract nucleus. Applying this threshold, the mask of nucleus (Nucleus Mask) is obtained. Then, some morphological operations (Gonzalez and Woods, 2008[11]) are performed on the Nucleus Mask to clean up it. In this regard, at first, a morphological closing operator (with10-pixel-diameter disk) is used to join disconnected pixels. Next, a morphological opening operator (with 2-pixel-diameter disk) is applied to remove small objects connected to the mask. After that, a morphological hole-filling operator is utilized to fill inside of segmented regions. Usually, in the nucleus mask, there are some false objects; that in order to remove them, the regions which have the area lower than nucleus are disregarded. The result of applying nucleus segmentation has been presented in Figure 2g(Fig. 2) and the RGB image of Nucleus Mask has been shown in Figure 2h(Fig. 2). At the following, in order to split touching cells and obtaining the mask of each cell individually, the modified watershed transform based on gradient method (Bala, 2012[5]) is used (Figure 2i(Fig. 2)). Finally, the Nucleus Mask is subtracted from the Cell Mask to obtain the Cytoplasm Mask.

### B: Feature extraction 

In the next stage, some appropriate features are determined from the segmentation results of the nucleus and cytoplasm for each WBC. In this regard, after considering characteristics of all types of CML cells according to Table 1(Tab. 1), we calculated various image features similar to the previous work including: geometric, statistical, color and texture features. Then, in order to select the suitable features that able to properly discriminate each group of CML cell, we performed some feature selection methods by three statistical analyses including: one-way analysis of variance (ANOVA (Roscoe, 1975[24])), Principal component analysis (PCA (Smith, 2002[30])) and Boxplot (Frigge et al., 1989[8]). These statistical analyses are described as follows:

#### ANOVA

ANOVA is a statistical test used to compare mean values of variations of more than two groups. In this test, the discrimination capability of each individual feature is determined with a p-value, where a less p-value indicates that the corresponding feature can properly separate at least one group from others. In practice, p-value less than 0.05 are clinically considered significant (Acharya et al., 2014[1]). 

#### PCA

PCA is a statistical procedure that uses an orthogonal transformation to convert a set of features of possibly correlated variables into a set of features of linearly uncorrelated variables called principal components (PCs). To choose a reasonable number of PCs, we ran PCA with different number of PCs and computed a PC score each time. Finally, components including more than 99.9 % of the features were selected.

#### Boxplot

In descriptive statistics, a boxplot is a graphical representation of groups based on the five statistical features of each group. These five statistical features consist of minimum, maximum, median, lower quartile and upper quartile of data in each group. This compact graph is a proper way to summarize the distribution of each group of numerical data. Also, this graph can be used to determine the portion of overlapping groups. Moreover, it can indicate symmetry and skew in the data and identify outliers. The general structure of a boxplot shows in Figure 9a.

Our experimental results after performing PCA, ANOVA and boxplot on the typical features used in the previous papers show that they are not very appropriate features for classification of WBCs in CML cells. Therefore, in this work, we propose three new geometrical features and combined them with three typical image features to select them as the suitable features for classification. Three typical features include: 1- number of nucleus lobes (Num), 2- area of nucleus (AoN) and 3- average color of cytoplasm of b* component; also new geometrical features consist of: 1- minimum thickness of Nucleus Mask to minimum convex thickness of Nucleus Mask ratio (T1/T2), 2- minimum thickness of Nucleus Mask to Hausdorff distance (Huttenlocher et al., 1993[13]) between border of nucleus and Cell Mask (T1/HD) and 3- perimeter of smaller nucleus after splitting (P1).

### Typical features

#### 1-Number of nucleus lobes (Num) 

**Figure 3:**
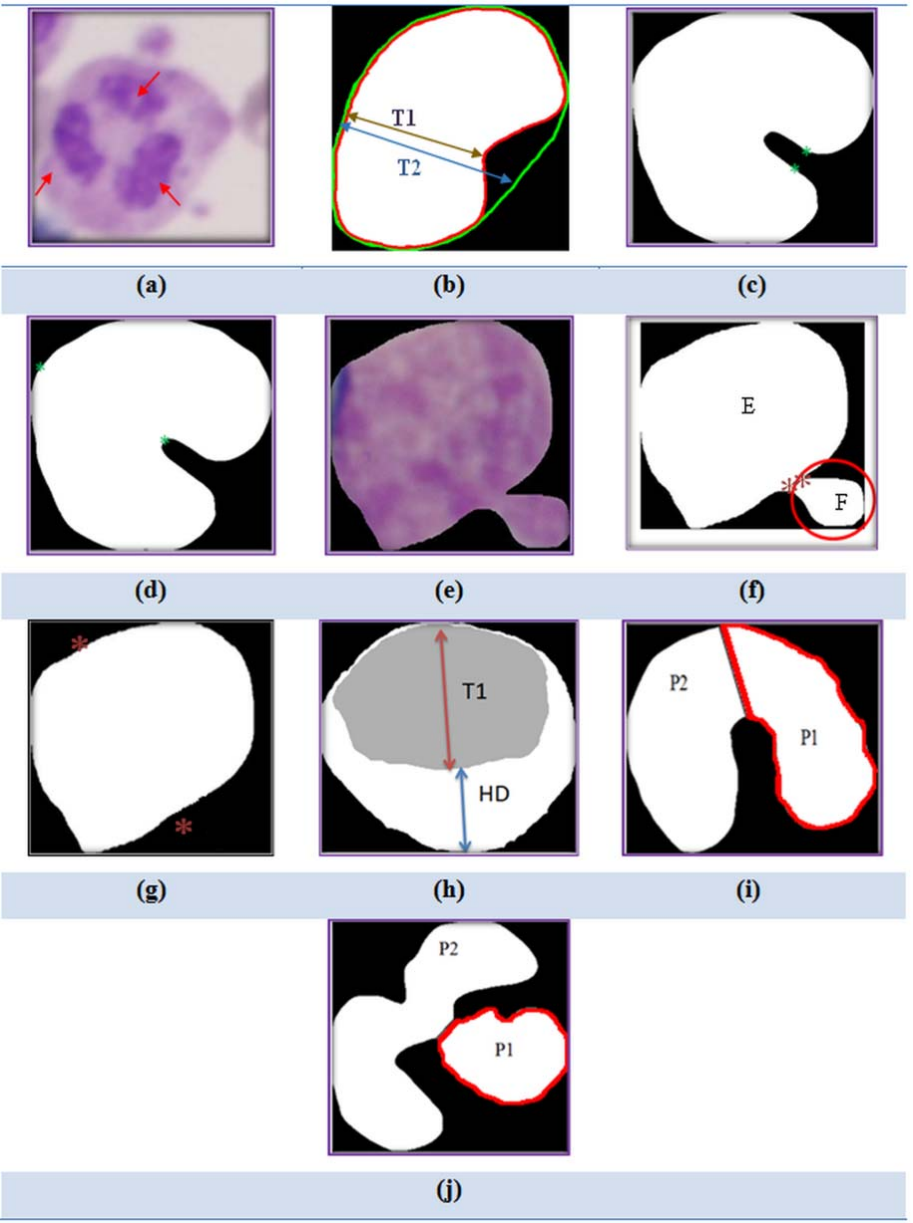
(a) Neutrophil with nuclear lobes, (b) Display T1 and T2, (c) A pair of points incorrectly determined for splitting by the original bottleneck algorithm, (d) Result of applying the proposed algorithm to correct the Step 1 of bottleneck algorithm, (e) RGB image of nucleus with an extra object, (f) Binary image of e, (g) Nucleus after removing extra object, (h) Display T1 and HD, (i) Binary image of Band nucleus after splitting, (j) Binary image of Neutrophil nucleus after splitting

After separation of the nucleus from a WBC, counting separated objects in the Nucleus Mask is determined by the number of nucleus lobes. Figure 3a(Fig. 3) shows one neutrophil cell with three lobes.

#### 2-Area of Nucleus (AoN) 

The area of an object in a binary (black and white) image can be defined by counting all white pixels inside the object (Nucleus).

#### 3-Average Color of Cytoplasm (ACoC)

As seen in Table 1(Tab. 1), cytoplasm's color varies during maturity stages. Thus, we compute the average color of cytoplasm in b* component of L*a*b* color space as an appropriate statistical feature. 

### New geometrical features

#### 1-Minimum thickness of Nucleus Mask to minimum convex thickness of Nucleus Mask ratio (T1/T2)

**Figure 4:**
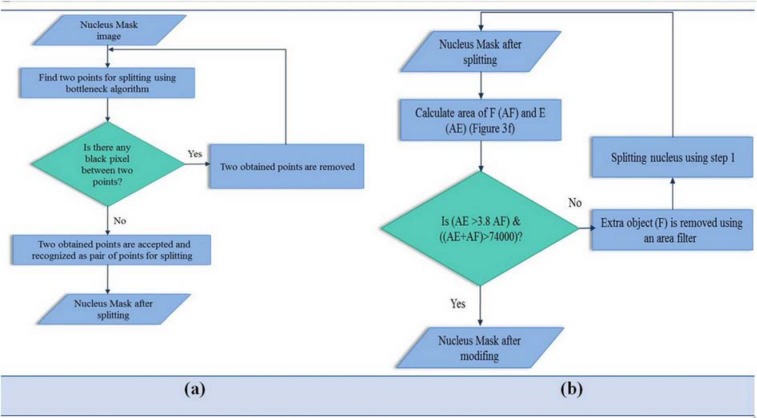
Block diagram of the proposed algorithm for modifying of bottleneck algorithm

Table 1(Tab. 1) shows that when the nucleus becomes more mature, the area of nucleus to area of cytoplasm ratio decreases, additionally, the shape of nucleus becomes more irregular (the shape of nucleus is neither round nor oval and has a notch). So, the minimum thickness of Nucleus Mask (T1) to the minimum convex thickness of Nucleus Mask (T2) ratio was used as a new geometrical feature. According to Figure 3b(Fig. 3), the red contour shows the perimeter of nucleus and green contour shows the perimeter of convex of Nucleus Mask. In this figure, T1 is indicated with the brown arrow and T2 is indicated with the blue arrow. We calculate this feature using bottleneck algorithm (Wang et al., 2012[35]). Bottleneck algorithm is based on two steps: 1- recognition of a pair of points for splitting, and 2- connect the candidate pair of points for splitting. For Step 1, in some cases (seen in Figure 3c(Fig. 3)), the original bottleneck algorithm incorrectly determines a pair of points for splitting. To overcome this problem, we present a novel algorithm (Figure 4a(Fig. 4)) to modify this method for finding correct points. According to the block diagram of this figure, we first apply Step 1 of the original bottleneck algorithm on the Nucleus Mask image. Next, we evaluate obtained points using a proposed technique. According to this technique, two obtained points are acceptable if there isn't a black pixel between them. Otherwise two points are removed and the bottleneck algorithm is employed again. The result of applying the proposed algorithm is shown in Figure 3d(Fig. 3). 

In the Step 2 of the bottleneck algorithm, sometimes the result of splitting process is not acceptable because of an extra object (F in Figure 3f(Fig. 3)), connected to the nucleus in the RGB image (Figure 3e(Fig. 3)). To solve this problem, we employ a novel algorithm, after splitting, shown as a block diagram in Figure 4b. As seen in this figure, where the area of the big nucleus is 3.8 times more than the small nucleus, and the area of two nucleus (AE+AF) is more than 74000 (pixels), splitting is acceptable. Otherwise, we remove small nucleus using an area filter, then apply the bottleneck algorithm again. Figure 3g(Fig. 3) displays the main nucleus and two points after using the proposed algorithm in order to split.

#### 2- Minimum thickness of Nucleus Mask to Hausdorff distance between nucleus and cell ratio (T1/HD)

Our experimental results demonstrate that minimum thickness of Nucleus Mask (T1) to Hausdorff distance (HD) between border of Nucleus Mask (U) and border of Cell Mask (V) ratio in three groups (Myeloblast, Promyelocyte and Myelocyte) of CML cells is different and we can use this feature as a worthy geometrical feature to classify these three groups (Figure 3h(Fig. 3)). Suppose *U* = {*u*_1_,*u*_2_,…,*u*_p_} and *V* = {*v*_1_,*v*_2_,…,*v*_p_} are two sets of points that determine border of Nucleus Mask and border of Cell Mask respectively. The distance between the point *u_i_* ∈ *U* and border *V* is defined as:


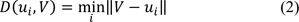



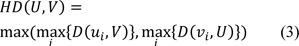


#### 3- Perimeter of smaller nucleus after splitting (P1)

The perimeter of an object can be defined by counting all of pixels in the border of it. After using the bottleneck algorithm, the perimeter of the smaller nucleus (P1) is calculated and it is used as a good geometrical feature for classification. Figure 3i and j(Fig. 3) show P1 in two different groups (Band and neutrophil) after splitting. 

### C: Classification

**Figure 5:**
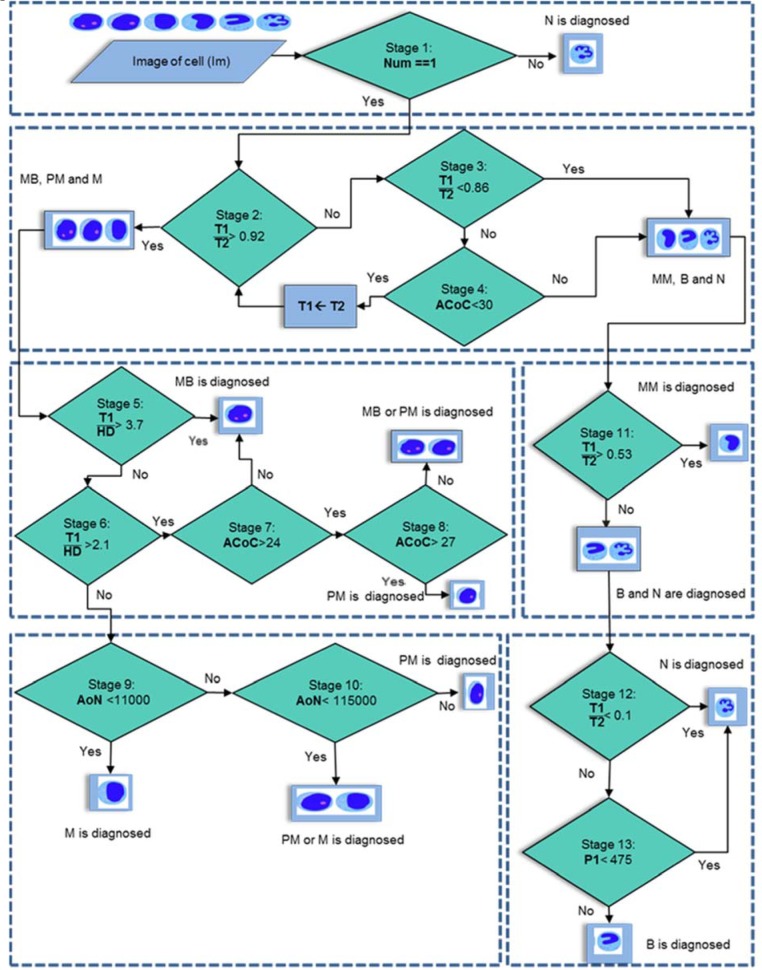
Block diagram of proposed decision tree classifier for classification of CML cells into 8 groups. Myeloblast (MB), Promyelocyte (PM), Myelocyte (M), Metamyelocyte (MM), Band (B), Neutrophil (N), Myeloblast or Promyelocyte (MB | PM) and Promyelocyte or Myelocyte (PM | M)

The main goal of this work is to classify the CML cells. To achieve this goal, we precisely analyzed all of the features obtained in the previous step and introduced a novel decision tree algorithm in order to cell classification. The block diagram of designed algorithm has been presented in Figure 5(Fig. 5). In this figure, threshold values have been set up after considering of all images in our training part of dataset. Also, our dataset includes some WBCs that two pathology experts of our research team couldn't surely classify them in a special group. Consequently, we considered two extra groups for these suspect WBCs and classified all of cells in our dataset into the eight groups. These groups are Myeloblast (MB), Promyelocyte (PM), Myelocyte (M), Metamyelocyte (MM), Band (B), Neutrophil (N), Myeloblast or Promyelocyte (MB | PM) and Promyelocyte or Myelocyte (PM | M). 

According to Figure 5(Fig. 5), in the Stage 1, for each cell image (Im), the Number of Nucleus (Num) is calculated to identify nucleus lobes. If the Num is greater than one, it will be recognized as a Neutrophil cell. Next in the Stage 2, T1/T2 is considered for detecting regular nuclei. So, upper values of 0.92 for this ratio are related to Myeloblast, Promyelocyte and Myelocyte. In contrast, lower values of 0.92 are related to irregular nuclei (Metamyelocyte, Band and Neutrophil). However, some nuclei in Myeloblast and Promyelocyte groups have notch similar to Metamyelocyte. So, a threshold is considered to separate Myeloblast and/or Promyelocyte from Metamyelocyte based on the color. In this regard, in the third stage, if T1/T2 for a nucleus is between 0.86 and 0.92; and ACoC is lower than 30 (Stage 4), the notch is not consider, instead, we consider the convex thickness of the Nucleus Mask (T2) instead of the irregular nucleus. Then, T2 is used as T1 because this value is used for next steps. After applying T1/T2 = 1, all of cells in Myeloblast, Promyelocyte, Myelocyte groups are distinguished from Metamyelocyte, Band and Neutrophil groups. Since the distance between border of nucleus and cell is different in these three groups and T1/HD can show this difference as well, the T1/HD is used for separating Myeloblast, Promyelocyte, Myelocyte groups. In this regard, a cell with T1/HD upper than 3.7 (Stage 5) is recognized as a Myeloblast cell. However, experimental observations show that T1/HD for some of cells in the Myeloblast group is lower than 3.7. For this type of cells, if T1/HD is more than 2.1 (Stage 6) and ACoC values less than 24, the cell is considered as a Myeloblast yet (Stage 7). In contrast, higher values of 27 are related to a Promyelocyte cell (stage 8), and a value between 24 and 27 is identified as a Promyelocyte or Myeloblast cell. In the next stage, in order to discriminate Myelocyte from Promyelocyte, the AoN feature is considered. AoN values lower than 110000 pixels are considered as an acceptable value for Myelocyte (stage 9). In contrast, higher values of 115000 pixels are related to Promyelocyte cell (stage 10). However, for AoN values between 110000 pixels and 115000 pixels, we can not separate Promyelocyte cells from Myelocyte cells. In the Stage 11, for separating the Metamyelocyte cells from Band and Neutrophil cells, if T1/T2 for a nucleus is between 0.53 and 0.86, the cell is diagnosed as a Metamyelocyte. Then, if the T1/T2 for a nucleus is lower than 0.1, the cell is diagnosed as a Neutrophil (Stage 12). Finally in the Stage 13, the P1 values upper than 475 pixels are related to Band and the P1 values lower than 475 pixels are related to Neutrophil. 

## Experimental Data and Validation Methods

### Evaluation parameters

For performance analysis, the classification results of our algorithms have been compared with diagnosed results of two experts using general evaluation parameters (Glas et al., 2003[10]) that are defined as follows:


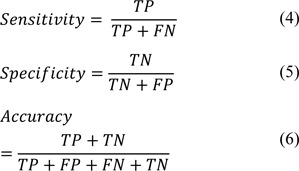


In these Equations, True positive (TP) indicates the cancerous cells identified correctly. False positive (FP) shows the non-cancerous cells identified as cancerous. False negative (FN) denotes the cancerous cells recognized as non-cancerous and when non-cancerous cells correctly recognized; they are specified by true negatives (TN). 

Also, in order to determine the level of agreement between the classification results of proposed computer-based method and diagnosis results of two pathologists, we have calculated Cohen's kappa coefficient (κ) (Momenzadeh et al., 2018[18]) as follows:


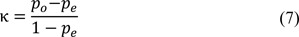


In Eq. (7) *p*_0_ is the actual observed agreement (identical to accuracy) and *p_e_* denotes chance agreement. They are defined as:


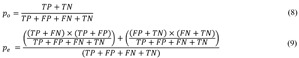


where κ = 1 shows the results of proposed algorithm and expert's opinion have perfectly agreed together, while κ = 0 indicates outcomes of proposed method and expert's opinion do not agree with each other.

### Experimental data

**Figure 6:**
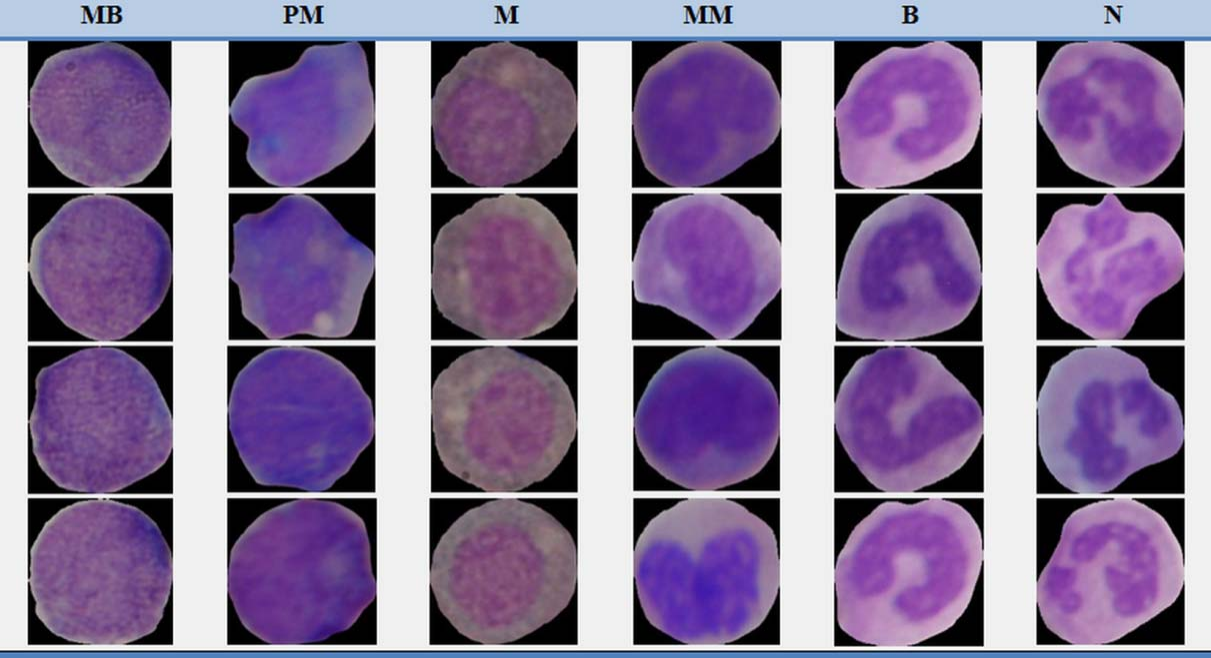
Sample cells of non-cancerous bone marrow aspirations from our dataset

**Figure 7:**
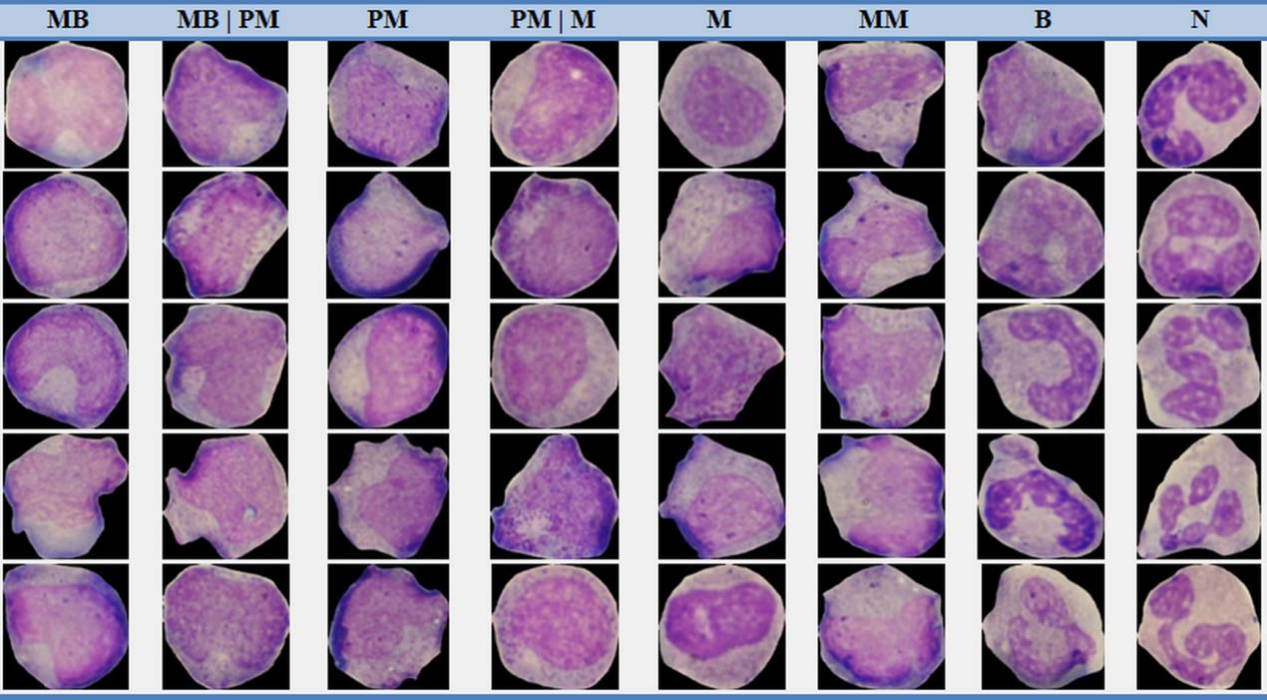
Sample cells of cancerous peripheral blood smear from our dataset

In this research, blood samples were provided from 6 non-cancerous bone marrow aspirations and 19 cancerous peripheral blood samples. Images were obtained from microscope slides by a light microscope after smeared and stained by Gismo. The images of our dataset were captured with a Nikon1 V1 camera mounted on Nikon Eclipse 50i microscope with a magnification of 1000. A total number of 580 images were gathered for our dataset. All images are in RGB color space and saved in the JPG format with 2592 × 3872 pixels. The images were prepared by pathologist experts in the Al-Zahra hospital and Omid hospital, Isfahan, Iran. For validation of our method, we selected 1730 WBCs from the dataset and asked two experts (E1 and E2) in order to manually determine the border of WBCs and separately label cells. Sample cells of non-cancerous bone marrow aspirations as well as sample cells of cancerous peripheral blood smear from our dataset have been shown in Figure 6(Fig. 6) and Figure 7(Fig. 7) respectively.

## Results and Discussion

In this project, our dataset was provided in several phases during more than one year. In this regard, the feature selection and parameter setting in the decision tree classifier were performed on only 95 cells of non-cancerous bone marrow aspiration and 192 cells of cancerous peripheral blood smear. The remaining 1443 cells including 619 WBCs of non-cancerous bone marrow aspiration and 824 CML cells of the cancerous peripheral blood smear were utilized for testing and generalizing.

To achieve the desired results, we planned and performed our experimental procedure in five steps as follows: 

***Step 1:**** Classification of non-cancerous bone marrow aspirations using typical features and SVM Classifier *

In the first step, 119 WBCs of non-cancerous bone marrow aspirations were considered. After considering of the previous papers individually (Putzu et al., 2014[21]; MoradiAmin et al., 2016[19]; Saeedizadeh et al., 2016[28]; Rezatofighi and Soltanian-Zadeh, 2011[23]; Sarrafzadeh et al., 2014[29]) in order to improve the classification results, we combined features of these papers and extracted finally 33 typical features. These features included eleven geometric features of in CML cells (1- area of cell, 2- area of nucleus, 3- nucleus area to cell area ratio, 4- perimeter of cell, 5- perimeter of nucleus, 6- solidity of nucleus, 7- eccentricity of nucleus, 8- minor axis length of nucleus, 9- elongation of nucleus, 10- form factor of nucleus, 11- the average of Fourier coefficients), six statistical features of cell obtained from G component of RGB color space (12- mean , 13- standard deviation , 14- smoothness, 15- uniformity , 16- third central moment and 17- entropy) and six statistical features of nucleus from the G component of RGB color space (18- mean, 19- standard deviation, 20- smoothness, 21- uniformity, 22- third central moment and 23- entropy), six statistical features of cytoplasm calculated from the b* component of L*a*b* color space (24- mean, 25- standard deviation, 26- smoothness, 27- uniformity, 28- third central moment and 29- entropy); also two texture features based on GLCM matrix of nucleus (30- contrast and 31- corrolotion) and two texture features based on GLCM matrix of cytoplasm (32- contrast and 33- corrolotion) from b* component of L*a*b* color space.

After that, the SVM classifier was utilized for categorizing of the cells. SVM is one of the powerful classification methods that typically are used in the previous work. For this study, according to (Saeedizadeh et al., 2016[28]) a radial basis functions kernel with sigma = 3 was used for SVM classifier. To evaluate the classification results, we divided our primary dataset into 80 % for training and 20 % for testing. Also, for validation, we used K-fold cross-validation with K = 10. Then, the evaluation parameters based on the outcomes of the classifier and the joint opinion of expert 1 and expert 2 (joint (E1, E2)) were calculated that the average values of specificity, sensitivity and accuracy were 56 %, 78 % and 76 % respectively. 

**Figure 8:**
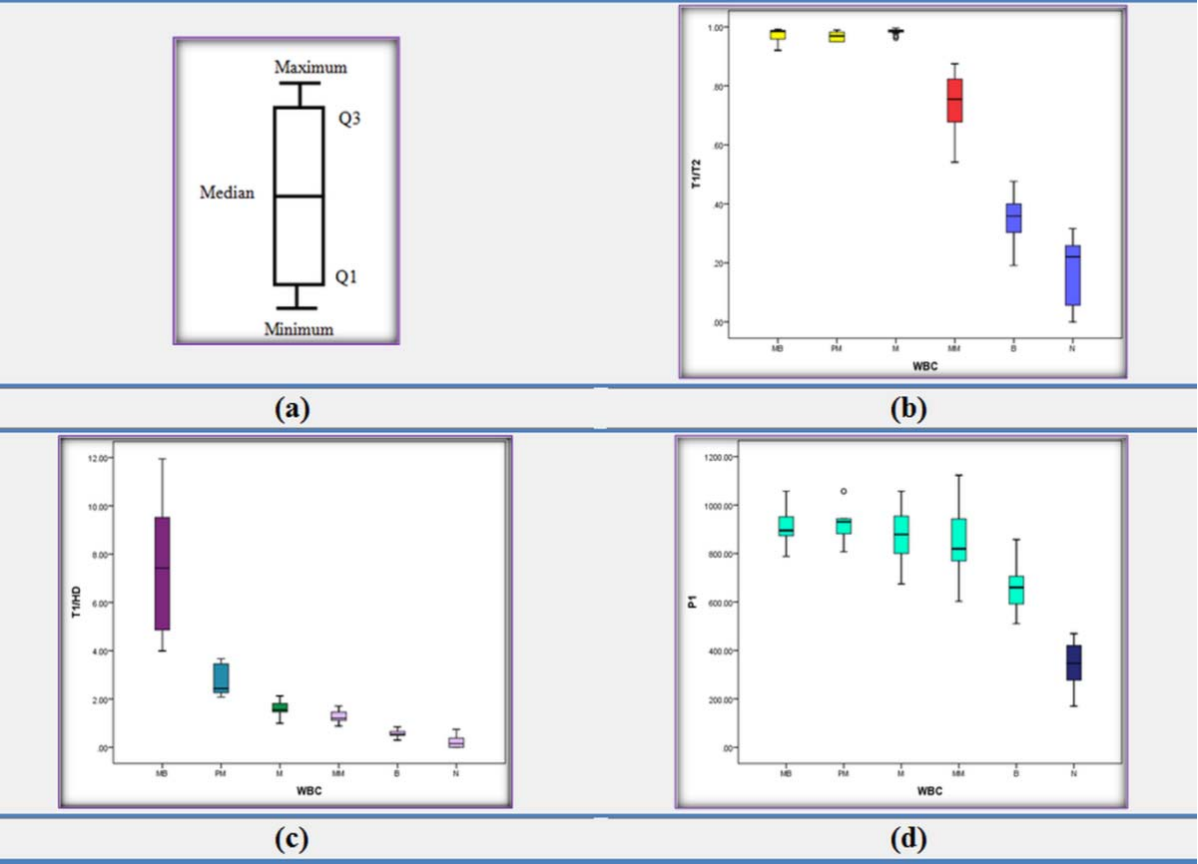
Result of simulation in step 1: (a) Plot between feature index and p-value for showing feature significance and (b) PCA performance based on number of principle components

At the following, we exploited feature selection methods to remove correlation between features, reduce the feature vector size and increase the performance of the classifier. In this regards, ANOVA and PCA methods were applied to select the suitable features for classification. For this purpose, at first, feature selection using ANOVA was used. The results of this method are portrayed as a plot between feature index and p-value in Figure 8a(Fig. 8). This figure can indicate significant features that able to discriminate different groups. The features with p-values less than 0.05 can be used to discriminate the six classes with higher accuracy. Using of ANOVA, we found that 26 features from the 33 features are statistically significant; and are capable to classify myeloid series into Myeloblast, Promyelocyte, Myelocyte, Metamyelocyte, Band and Neutrophil classes. 

Then, we employed the SVM classifier that the average values of sensitivity, specificity and accuracy for the six groups were obtained 60 %, 93 % and 88 % respectively. These evaluation parameters revealed that applying only ANOVA method does not lead to the desired classification results. 

Following this step, the PCA technique was used on 26 selected features after ANOVA test. To choose a reasonable number of PCs, PCA was run with a different number of PCs ranging from 1 to 12; each time, a PC score was computed. According to Figure 8b(Fig. 8), a PC score for 12 features of the 26 features include more than 99.9 % of features and can be an adequate choice for classification of the six groups of CML cells. After using SVM classifier on these 12 features, average values of 87 % for sensitivity, 97 % for specificity and 96 % for accuracy were achieved for the six groups. The evaluation parameters show that applying PCA after ANOVA can improve the results of classification, however the sensitivity has not been satisfactory yet. 

***Step 2:**** Classification of non-cancerous bone marrow aspirations using proposed features and SVM classifier*

In this step, we attempted to introduce a few reliable set of features that better distinguish each type of CML cells. For this propose, we evaluated various number of features, used in the previous work, and some proposed new features by boxplot. Next, by comparing and considering these boxplots, we selected four proposed features (T1/T2 feature, T1/HD feature, P1 feature and number of nucleus lobes (Num)) as the suitable features for classification of CML cells. 

**Figure 9:**
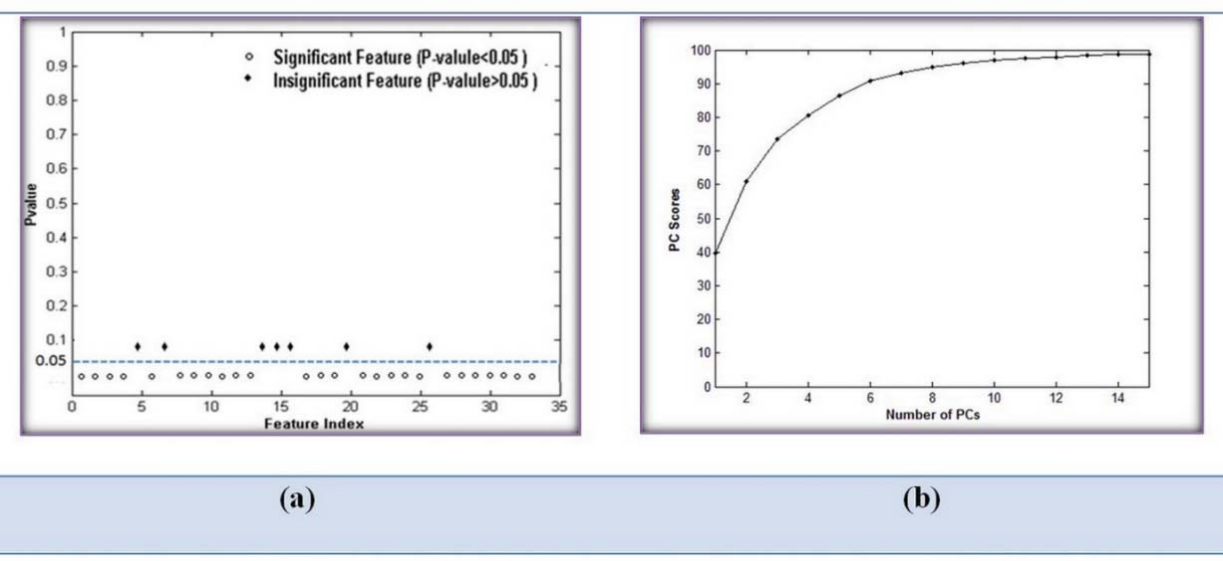
Display of the best features using boxplot for CML cell classification of non-cancerous bone marrow aspirations. (a) Description of a box plot, (b) "T1"/"T2" (the minimum thickness of Nucleus Mask to minimum convex thickness of Nucleus Mask ratio), (c) "T1"/"HD" (the minimum thickness of Nucleus Mask to minimum convex thickness of Nucleus Mask ratio), (d) P1 (perimeter of smaller nucleus after splitting)

The results of selected features using the box plot have been displayed in Figure 9b-d(Fig. 9). As observed from the box plots of Figure 9b(Fig. 9), there are significant differences using the T1/T2 feature, among three groups (Myeloblast, Promyelocyte and Myelocyte), Metamyelocyte group and two groups (Band and Neutrophil). Also, the T1/HD box plots (Figure 9c(Fig. 9)) show obvious differences among Myeloblast, Promyelocyte and Myelocyte. Furthermore, as shown in the box plots of Figure 9d(Fig. 9), there is discrimination between Band and Neutrophil in the P1 feature. Moreover, Neutrophile cells can be recognized where number of nucleus lobes were more than one (Num>1).

The SVM classifier was then applied for classification of the six groups of CML cells and achieved the average values of specificity, 92 %, sensitivity, 98 %, and accuracy, 97 %. The evaluation parameters show that applying proposed features have improved the results of classification using fewer numbers of features.

***Step 3:**** Classification of non-cancerous bone marrow aspirations using proposed features and new decision tree classifier*

In this step, as in the previous step, we used 119 CML cells of non-cancerous bone marrow aspiration and four proposed features. However, in order to obtain the desired results, we employed the new decision tree classifier, presented in the previous section. In the training stage, for 95 cells, we set up all thresholding values for the classifier in order to obtain the best results (100 % for the specificity, sensitivity and accuracy). After achieving the desired results, we tested our algorithm on 24 WBCs and compared the classification results to the joint opinion of two experts that the average values of specificity, sensitivity and accuracy were obtained 100 %, 100 % and 100 % respectively.

At the following, for generalization, we applied our algorithm on a new dataset including 595 CML cells of non-cancerous bone marrow aspiration and compared the classification results to the joint opinion of two experts. The average values of specificity, sensitivity and accuracy are attained 96 %, 98 % and 98 % respectively. Moreover, Cohen's kappa coefficient between joint (E1, E2) and proposed automatic algorithm (Auto_alg) was achieved 0.97.

***Step 4:**** Classification of cancerous peripheral blood smear using proposed features and new decision tree classifier *

**Figure 10:**
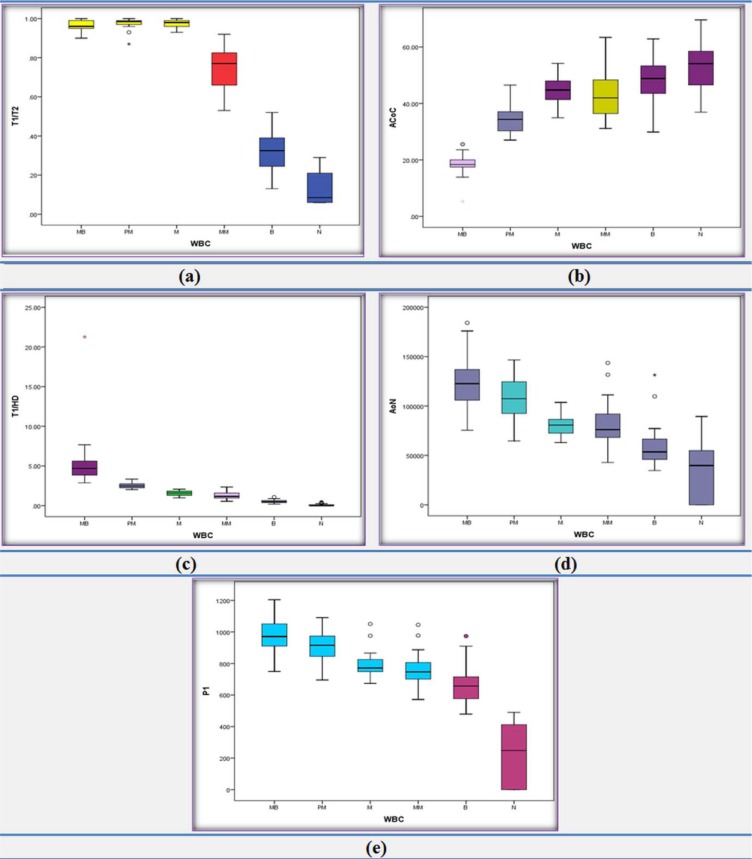
Display of the best features using boxplot for classification of CML cells. (a) "T1"/"T2" (the minimum thickness of Nucleus Mask to minimum convex thickness of Nucleus Mask ratio), (b) ACoC (average color of cytoplasm, (c) "T1"/"HD" (the minimum thickness of Nucleus Mask to minimum convex thickness of Nucleus Mask ratio), (d) AoN (area of nucleus) and (e) P1 (perimeter of smaller nucleus after splitting)

Since, the acceptable results for the classification of non-cancerous bone marrow in the step 3 were obtained; in this step, we employed the same four proposed features and the same decision tree classifier, used in previous step, on 240 CML cells of the cancerous peripheral blood smear. The average values of sensitivity, specificity and accuracy for the six groups using our classifier were obtained 89 %, 92 % and 91 % respectively. Then, in order to achieve better results, at first, we attempted to modify the parameters of the classifier, but the results did not substantially change. Next, we added two features (ACOC and AON) to our feature set. The results of selected features using box plot have been displayed in the Figure 10a-e(Fig. 10). 

As seen in the box plots of Figure 10a(Fig. 10), there are substantial differences using the T1/ T2 feature, between three groups (Myeloblast, Promyelocyte and Myelocyte) and other groups (Metamyelocyte, Band and Neutrophil). The ACoC box plots (Figure 10b(Fig. 10)) indicates considerable variations between Myeloblast, Promyelocyte and Metamyelocyte; so this feature can be used for separating them. Also, T1/HD box plots (Figure 10c(Fig. 10)) show a notable difference between Myeloblast and Promyelocyte and AoN box plots (Figure 10d(Fig. 10)) can be employed for separating Promyelocyte and Myelocyte. Furthermore, from the box plots of Figure 10e(Fig. 10), we can find clear discrimination between Band and Neutrophil in the P1 feature.

Additionally, we added two extra groups to the six main groups, because our experts' investigation revealed that in this series of cells, there can be an overlap between Myeloblast and Promyelocyte (MB|PM); also an overlap between Promyelocyte and Myelocyte (PM | M). 

Finally, in order to set up classifier parameters related to two new features and two extra groups, in the training stage, for 192 cancerous cells, we tuned the related thresholding values of the classifier to get the best results (100 % for the specificity, sensitivity and accuracy). After reaching the appropriate results, we tested our algorithm on 48 remaining WBCs of cancerous cells and compared the classification results to the joint opinion of two pathologists that the average values of specificity, sensitivity and accuracy were achieved 100 %, 100 % and 100 %, respectively, for the eight groups of the CML cells.

**Figure 11:**
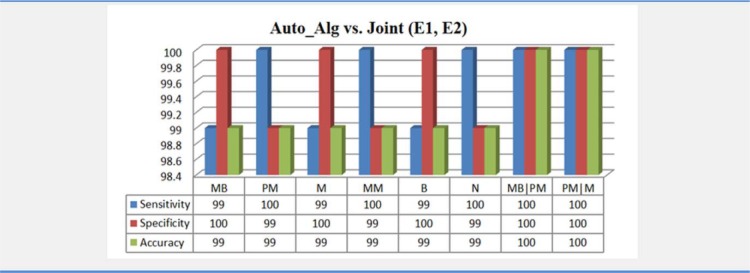
Results of automatic algorithm (Auto_alg) versus joint opinion between Expert1 and Expert2 (joint (E1, E2)) for classification of cancerous peripheral blood smear. MB (Myeloblast), PM (Promyelocyte), M (Myelocyte), MM (Metamyelocyte), B (Band) and N (Neutrophil)

After that, for generalization, we utilized the proposed classification method on a new data including 776 CML cells of the cancerous peripheral blood smear and compared the classification results to the joint opinion of two experts. The results of the automatic algorithm versus joint (E1, E2) have been shown in the Figure 11(Fig. 11). According to this figure, calculated performance parameters for all of 8 groups are above 99 %. Also, Cohen's kappa coefficient between joint (E1, E2) - Auto_alg pairing is attained 0.99. These worthy results confirm validity of proposed algorithm to classify CML cells of the cancerous peripheral blood smear as well.

***Step 5:**** Classification of both non-cancerous bone marrow aspirations and cancerous peripheral blood smear using proposed features and new decision tree classifier*

In the final step, we utilized proposed decision tree classifier (Figure 5(Fig. 5)) on all of our testing set (1443 cells) including 619 WBCs of non-cancerous bone marrow aspiration and 824 CML cells of the cancerous peripheral blood smear to classify them into the eight groups. 

**Figure 12:**
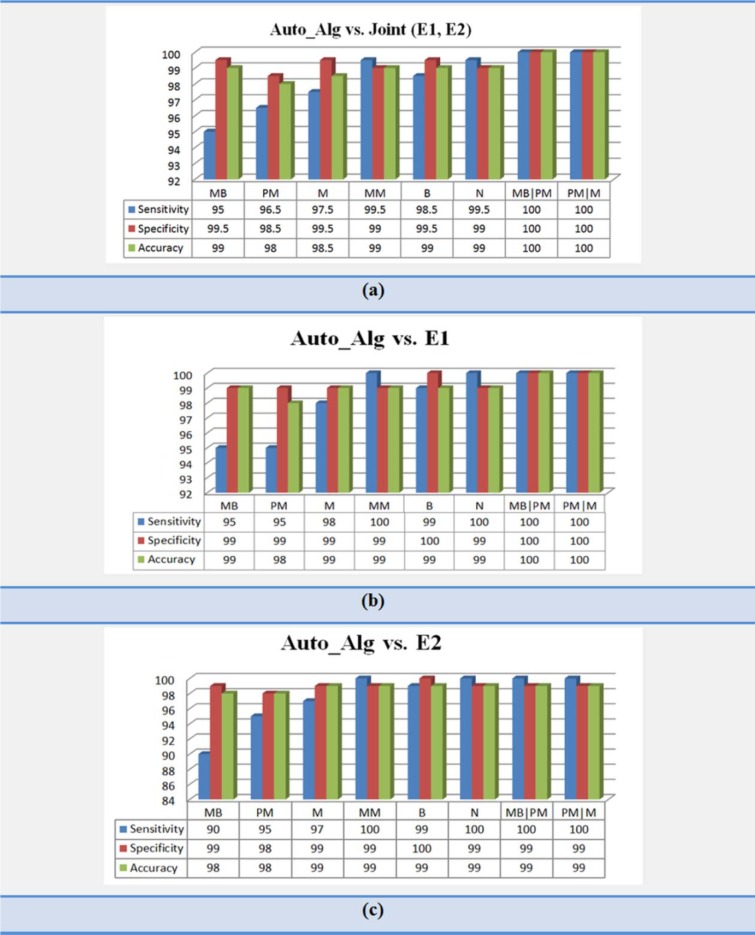
Results of automatic algorithm (Auto_alg) for classification of both non-cancerous bone marrow aspirations and cancerous peripheral, versus (a) Joint opinion between Expert1 and Expert2 (Joint (E1, E2)), (b) Opinion of Expert1 and (c) Opinion of Expert2. MB (Myeloblast), PM (Promyelocyte), M (Myelocyte), MM (Metamyelocyte), B (Band) and N (Neutrophil)

After classification, to validate the effectiveness of the proposed algorithms, we calculated sensitivity, specificity and accuracy as the performance measures between the manually labeling (E1, E2 and joint (E1, E2)) and the results obtained by the proposed classifier (Auto_alg) for our dataset. These evaluation parameters for Auto_alg versus joint (E1, E2), Auto_alg versus E1 and Auto_alg versus E2 have been shown in Figure 12a(Fig. 12), Figure 12b(Fig. 12) and Figure 12c(Fig. 12) respectively. The results between Auto_alg and joint (E1, E2), show that the average values of accuracy, specificity and sensitivity respectively have been 99 %, 99.4 % and 98.3 % for all of the eight groups. Consequently, as can be seen from Figure 12(Fig. 12), the results of our method verify reliability and validation of proposed algorithm to classify CML cells. 

Furthermore, in order to determine the level of agreement between the classification results of proposed method and the manually labeling by two pathologists (E1, E2 and joint (E1, E2)), we computed the Cohen's kappa coefficient. Moreover, this coefficient was determined between two experts (E1 and E2). The obtained Cohen's kappa coefficients are presented in Table 2(Tab. 2). According to this table, Cohen's kappa coefficient has been 0.97 between E1 and E2 pairing while between Auto_alg and joint (E1, E2) pairing is very close to 1 (0.99). Also, this coefficient demonstrates level of agreement between the automatic algorithm and Expert 1 (0.98) is more than the automatic algorithm and Expert 2 (0.97). As a result, calculated Cohen's kappa coefficients verify conformity between the result of the automatic algorithm and two experts.

## Conclusions

Semi- or fully-automatic analysis of microscopic images can be a valuable tool to assist pathologist to diagnose blood diseases. One of the main challenges in automatic processing of microscopic images is classification of WBCs. In this paper, we proposed a simple and efficient method for classification of effective cells in CML. In the proposed method, at first, WBCs were segmented from captured and prepared microscopic images of blood samples. Next, a new combination of typical and proposed features were extracted from neutrophils series. The novel designed tree classifier was then applied on these extracted features to categorize cells into the eight groups. Finally, to obtain the desired results, a five-step procedure was performed. In each step, the classification results of proposed method were compared to the opinions of two experts using different performance metrics including sensitivity, specificity and accuracy. The calculated performance metrics show that the results of our approach are highly similar to the results of expert's diagnosis. Moreover, Cohen's kappa coefficient as a statistical index was calculated that indicated reliability of our classification method and verified their high agreement with the diagnosis results of two pathologists. According to these worthy results, it can be concluded that the proposed technique has high ability in classification of CML cells and can be employed as a suitable tool for helping to the pathologists for diagnosis of CML disease. 

## Acknowledgements

The authors would like to thank the Pathology department of Al-Zahra and Omid hospital, Isfahan, Iran for providing our dataset.

## Conflict of interest

The authors declare that they have no conflict of interest.

## Ethical approval

This study was approved by the ethics committees of Isfahan University of Medical Sciences (Iran) under grant number 394325. All the subjects were given informed written consent prior to participation in the study.

## Figures and Tables

**Table 1:**
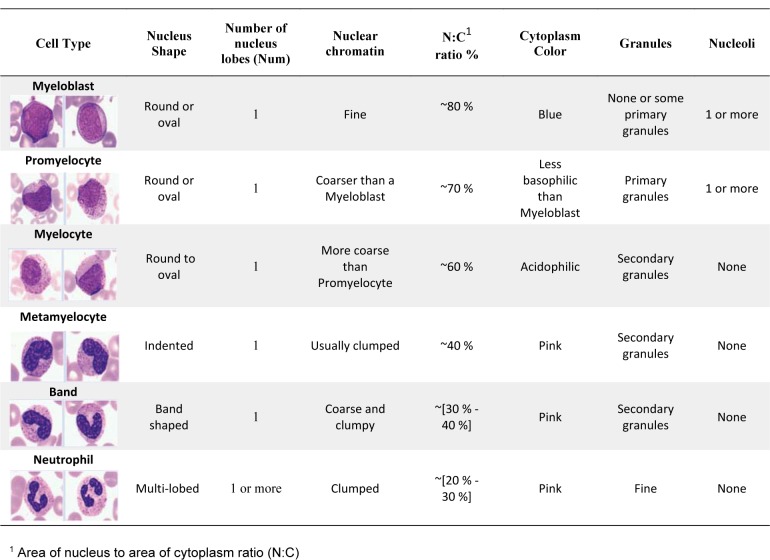
Characteristics of all types of myeloid cells in the maturation stages

**Table 2:**
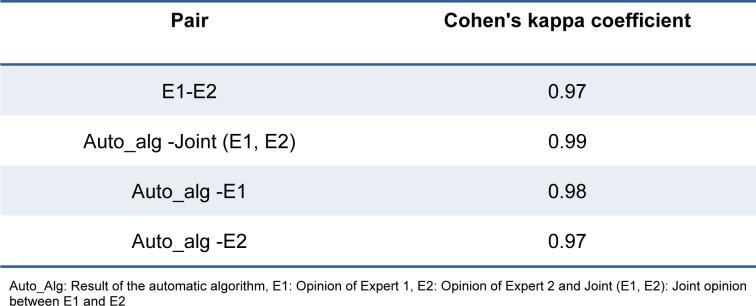
Calculated Cohen's kappa coefficients for determining level of agreement
